# Microvascular injury and hypoxic damage: emerging neuropathological signatures in COVID-19

**DOI:** 10.1007/s00401-020-02190-2

**Published:** 2020-07-08

**Authors:** Zane Jaunmuktane, Ula Mahadeva, Anna Green, Vivek Sekhawat, Nicholas A. Barrett, Lucy Childs, Manu Shankar-Hari, Maria Thom, Hans Rolf Jäger, Sebastian Brandner

**Affiliations:** 1 Division of Neuropathology, National Hospital for Neurology and Neurosurgery, University College London NHS Foundation Trust, London, UK; 2 Department of Clinical and Movement Neurosciences and Queen Square Brain Bank for Neurological Disorders, Queen Square Institute of Neurology, University College London, London, UK; 3 Department of Cellular Pathology, Guy’s and St Thomas’ NHS Foundation Trust, London, UK; 4 Department of Intensive Care Medicine, Guy’s and St Thomas’ NHS Foundation Trust, London, UK; 5 Faculty of Life Sciences and Medicine, King’s College London, London, UK; 6 Department of Radiology, Guy’s and St Thomas’ NHS Foundation Trust, London, UK; 7 School of Immunology and Microbial Sciences, King’s College London, London, UK; 8 Department of Clinical and Experimental Epilepsy, Queen Square Institute of Neurology, University College London, London, UK; 9 Lysholm Department of Neuroradiology, National Hospital for Neurology and Neurosurgery, University College London NHS Foundation Trust, London, UK; 10 Neuroradiological Academic Unit, Department of Brain Repair and Rehabilitation, Queen Square Institute of Neurology, University College London, London, UK; 11 Department of Neurodegenerative Disease, Queen Square Institute of Neurology, University College London, London, UK

In patients with COVID-19, neurological complications are increasingly recognised, but only few neuropathological studies are available, documenting microthrombi and acute infarcts [1], hypoxic changes with no specific pathology [9] or perivascular lymphocytic infiltration in brainstem [10]. We report here neuropathology of two COVID-19 patients with findings in one strikingly similar to those described in a recent case report, with neocortical infarcts and small haemorrhagic and non-haemorrhagic white matter lesions [8], suggesting an emerging pattern of characteristic alterations, also observed radiologically [3].

Clinical data for both patients are provided in supplementary Fig. 1 and neuropathology and imaging in Figs. 1, 2 and supplementary Figs. 2, 3, 4.

**Fig. 1:**
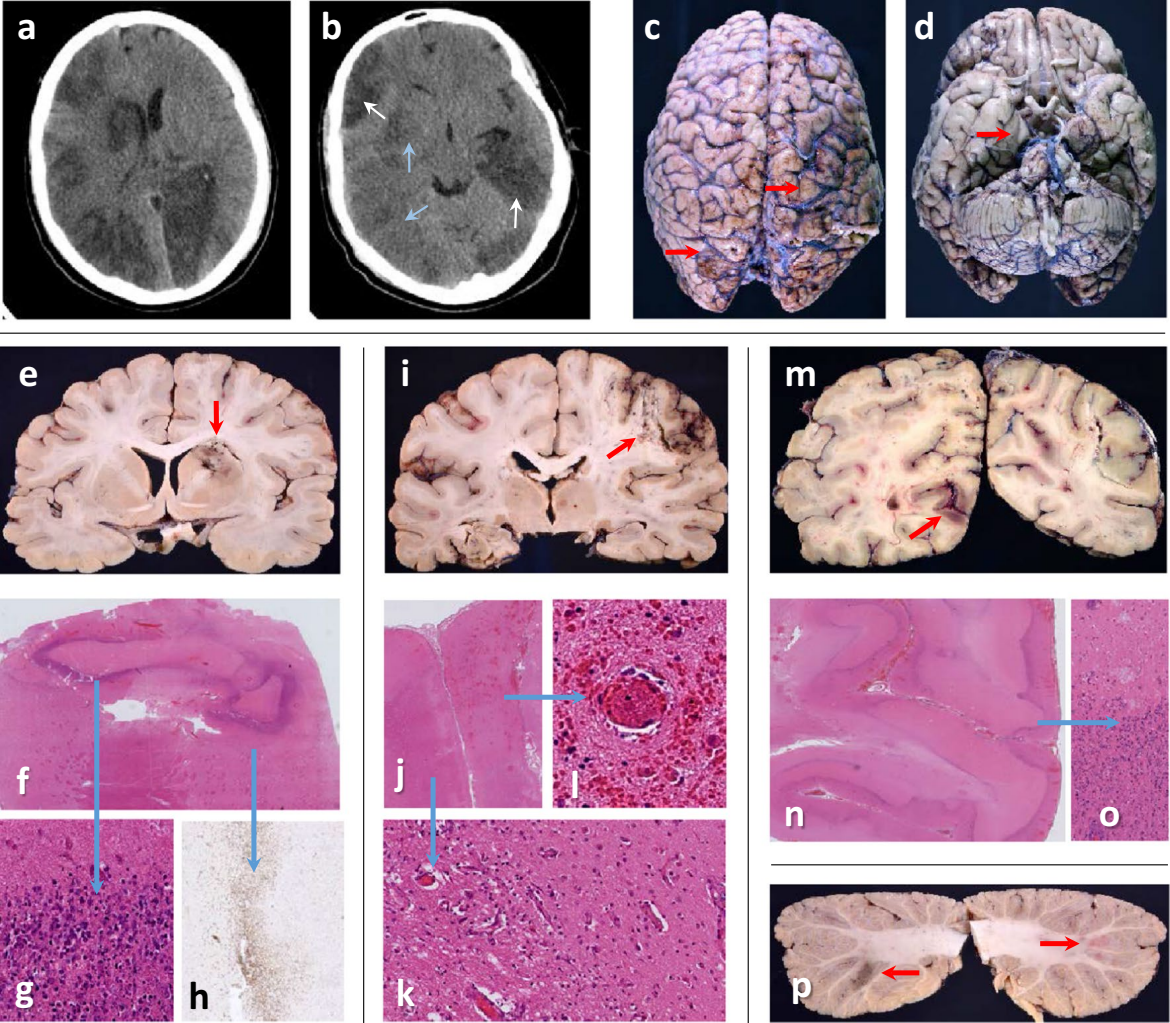
Case 1: **a**, **b** Head-CT shows recent (blue arrows) and established (white arrows) multifocal infarcts. **c** Rusty discolouration (red arrows) on brain surface. **d** Right uncal grooving (red arrow). **e** Bilateral acute and subacute watershed infarcts in the anterior-MCA and MCA–PCA territories and a subacute infarct in the right lentiform nucleus (red arrow). **f, g** Dense inner rim of degenerating neutrophils (blue arrow) and **h,** an outer rim (blue arrow) of macrophages (CD68). **i, j** Macroscopy and microscopy of confluent infarcts across the right MCA territory (acute and subacute, red arrow). **k** Infarcts are ischaemic, with granulation tissue and macrophages (blue arrow), or **l,** with perivascular haemorrhages and fibrin thrombi (blue arrow). **m** Bilateral acute and subacute infarcts in the PCA territories (both occipital lobes (red arrow), and **i** left hippocampus and thalamus. **n, o** Frequently, subacute infarcts show prominent leukocytoclastic reaction (blue arrow). **p** Multiple subacute cortical infarcts in both cerebellar hemispheres (red arrows)

**Fig. 2:**
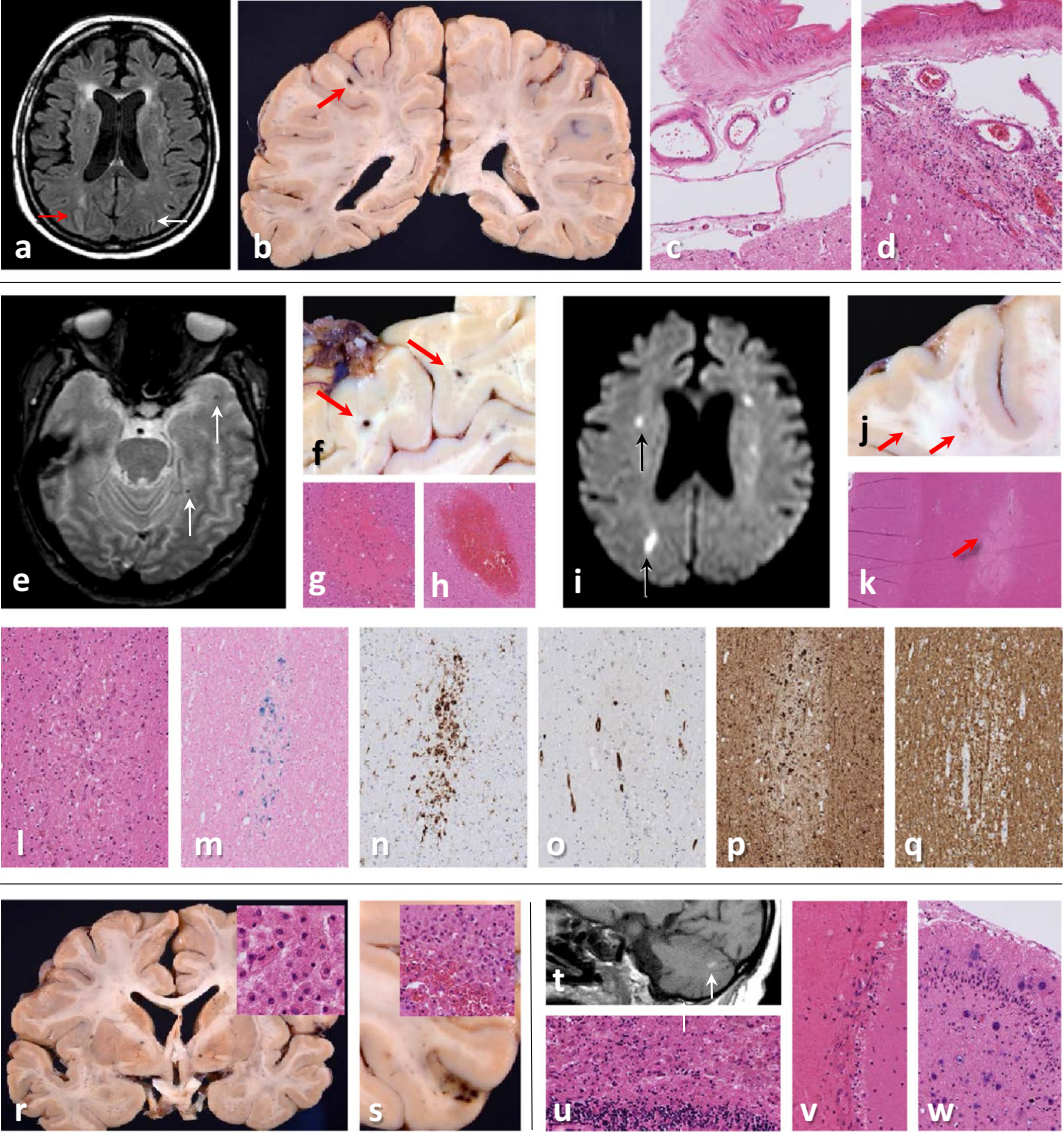
Case 2: **a** MRI brain (FLAIR image) shows leukoaraiosis and high signal intensity in the right (red arrow) but not left (white arrow) intraparietal sulcus. **b** Microhaemorrhage on the left (red arrow), but no gross leptomeningeal pathology. **c, d** Histology shows mild leptomeningeal lymphohistiocytic inflammation in the right (**d**), but not left (**c**) intraparietal sulcus. **e** Arrows point to microhaemorrhages (T2*weighted MRI), **f** macroscopically (red arrows) and **g, h** microscopically corresponding to acute and subacute white matter microbleeds. **i** Small acute infarcts (DWI MRI, black arrows), **j** macroscopically corresponding to white matter lesions (ø1-4 mm, red arrows). **k** Histologically, some are classic acute and subacute microinfarcts (red arrow), **l** whilst others contain haemosiderin-laden macrophages, **m** shown with Perls staining. **n** CD68 accentuates many more lesions than evident macroscopically or on MRI. **o** Unremarkable blood vessels are seen within some of the white matter microlesions (CD34 immunostaining). **p** Swollen axons (indicating damage) on neurofilament (SMI31) immunostaining **q** but no demyelination (myelin basic protein (SMI94) immunostaining). **r** Macroscopic and microscopic examination (inset in **r** shows macrophage-rich necrosis) reveal bilateral subacute pallidal infarcts. **s** Occasional subacute microinfarcts in the cortex (ø1–5 mm), some with haemorrhagic transformation (inset in **s**). **t** High MRI T1 signal foci (white arrow) in the cerebellum correspond histologically to **u** subacute infarct, **v** fresh leptomeningeal haemorrhages and (not shown) non-haemorrhagic white matter microlesions. **w** Several chronic infarcts and microinfarcts (likely embolic) in cerebellar cortex and right thalamus (not shown)

Patient 1, a male in his fifties, suffered from cardiac arrest, shortly before veno-venous extracorporeal membrane oxygenation (vvECMO) and succumbed to multifocal brain infarcts. The ischaemic lesions in watershed areas are in keeping with sustained hypotension during cardiac arrest. The exact cause of the large right middle cerebral artery (MCA) and the bilateral posterior (PCA) infarcts remains uncertain. Thromboembolic origin from the known pulmonary embolus is excluded due to closed foramen ovale. The infarcts may be due to local thrombosis or, similar to watershed infarcts, may have developed as a complication of protracted hypotension during cardiac arrest. The florid leukocytoclastic reaction in the infarcts may simply be a reaction to reperfusion injury but may also be due to augmented immune response.

The second patient, a female in her sixties, was intubated and ventilated, but after discontinuing sedation, remained unresponsive and died due to multiorgan failure. The bilateral pallidal infarcts most likely were caused by hypoxia. Possible pathogeneses for the cortical and white matter microlesions, including microbleeds, include viral infection-related vascular injury; immune-mediated; or hypoxia secondary to hypotension, local thrombosis, or thromboemboli. MRI-pathology correlation showed that leptomeningeal hyperintensity corresponded to lymphohistiocytic inflammation. Microglial nodules, neuronophagia and vascular injury, including signs of vasculitis, distant from the infarcts were not identified in either case and inflammation in the medulla was similar to patients with a variety of other neurological diseases (supplementary Table 1, supplementary Fig. 4).

Several mechanisms for the SARS-CoV-2-related neurological complications are plausible. First, direct viral invasion via haematogenous or retrograde axonal route with intracellular accumulation either in endothelial cells, smooth muscle cells, pericytes, inflammatory cells (particularly macrophages), neurones or glial cells. Second, an indirect process resulting from hypercoagulability-related thromboembolism or thrombus formation within the brain or an exaggerated cytokine/immune-mediated response to viral infection causing damage to blood vessel walls or cells in the brain.

Viral components specifically  in endothelial cells have been documented in kidney [7], lung and skin [5], but not with certainty in brain, although viral RNA of uncertain replicative and infective potential has been detected in the CSF [6] and brain tissue homogenates [7].

The few cases reported to date highlight the complexity of neuropathology in COVID-19 and the difficulty in untangling primary from secondary hypoxic/anoxic changes and iatrogenic aetiologies and suggest that a combination of different events, rather than a single mechanism, accounts for the various neurological complications, even within one patient.

It is striking that ACE2 expression is increased in ischaemic brains and also in blood vessels in patients with diabetes [2], given that ACE2 represents the receptor by which SARS-CoV-2 enters host cells. Similarly, certain treatment regimens, such as ECMO, may increase the risk of neurological complications [4]. We provide further neuropathological correlates to a radiological feature of subcortical white matter microvascular lesions, including microhaemorrhages. Through future neuropathological studies, it is hoped that the mechanisms leading to tissue damage in COVID-19 will continue to be elucidated, to enable timely and appropriate treatment options.

## Electronic supplementary material

Below is the link to the electronic supplementary material.

Supplementary file1 (PPTX 32177 kb)

Supplementary file2 (DOCX 35 kb)
