# Supplementary material for: Microvascular injury and hypoxic damage: emerging neuropathological signatures in COVID-19
Source: Acta Neuropathol. 2020 Jul 8;140(3):397–400. doi: 10.1007/s00401-020-02190-2 (PMC7340758; doi:10.1007/s00401-020-02190-2)
Supplement: Supplementary file 1 — Supplementary file1 (PPTX 32177 kb) [file 401_2020_2190_MOESM1_ESM.pptx]

## Slide 1
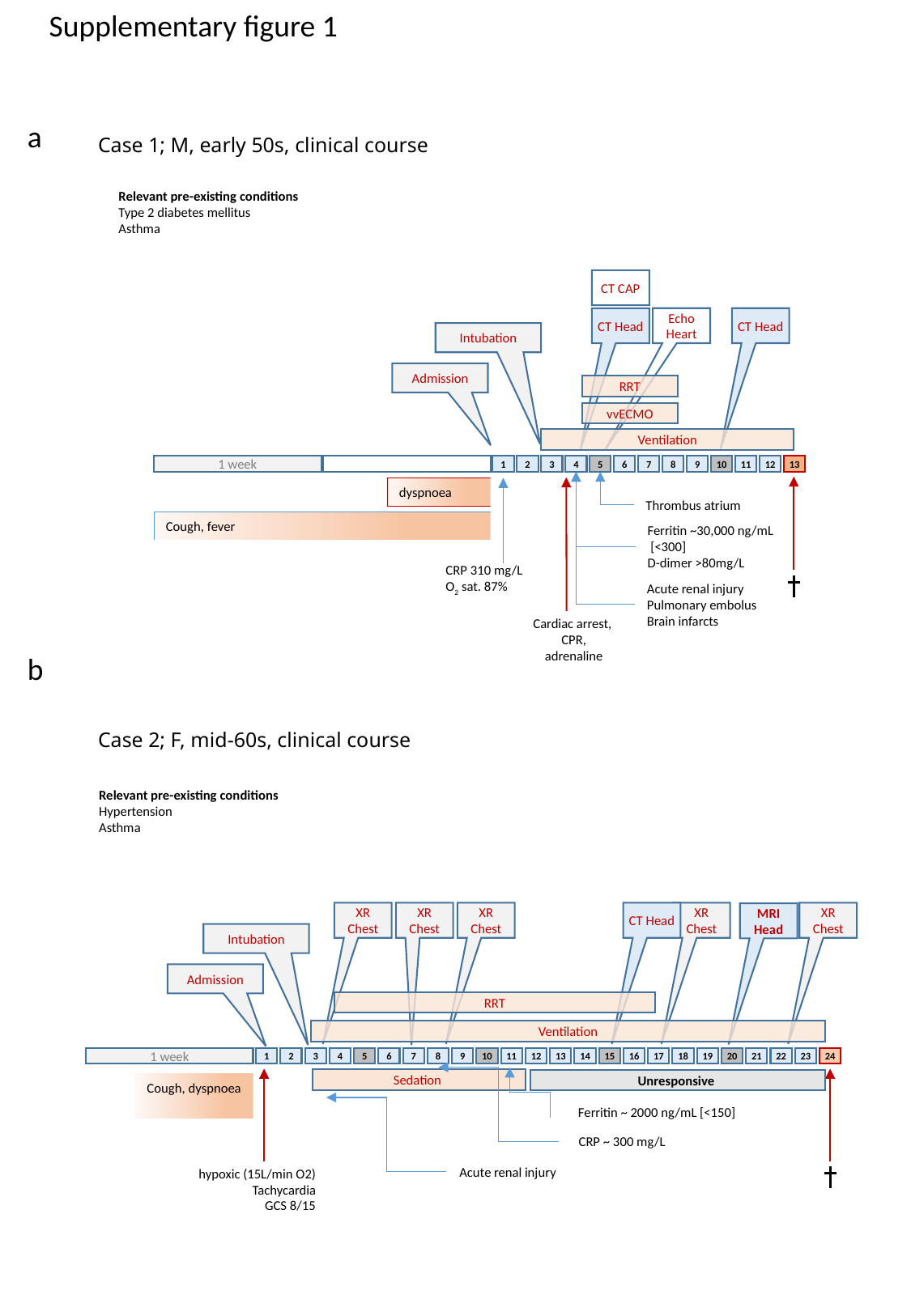

Supplementary figure 1
a
Case 1; M, early 50s, clinical course
Relevant pre-existing conditions
Type 2 diabetes mellitus
Asthma
CT CAP
Echo Heart
CT Head
CT Head
Intubation
Admission
RRT
vvECMO
Ventilation
1 week
1
2
3
4
5
6
7
8
9
10
11
12
13
dyspnoea
Thrombus atrium
Cough, fever
Ferritin ~30,000 ng/mL
 [<300]
D-dimer >80mg/L
CRP 310 mg/L
O2 sat. 87%
Acute renal injury
Pulmonary embolus
Brain infarcts
Cardiac arrest,
CPR,
adrenaline
b
Case 2; F, mid-60s, clinical course
Relevant pre-existing conditions
Hypertension
Asthma
XR Chest
XR Chest
XR Chest
CT Head
XR Chest
XR Chest
MRI Head
Intubation
Admission
RRT
Ventilation
1 week
1
2
3
4
5
6
7
8
9
10
11
12
13
14
15
16
17
18
19
20
21
22
23
24
Sedation
Unresponsive
Cough, dyspnoea
Ferritin ~ 2000 ng/mL [<150]
CRP ~ 300 mg/L
Acute renal injury
hypoxic (15L/min O2)
Tachycardia
GCS 8/15

## Slide 2
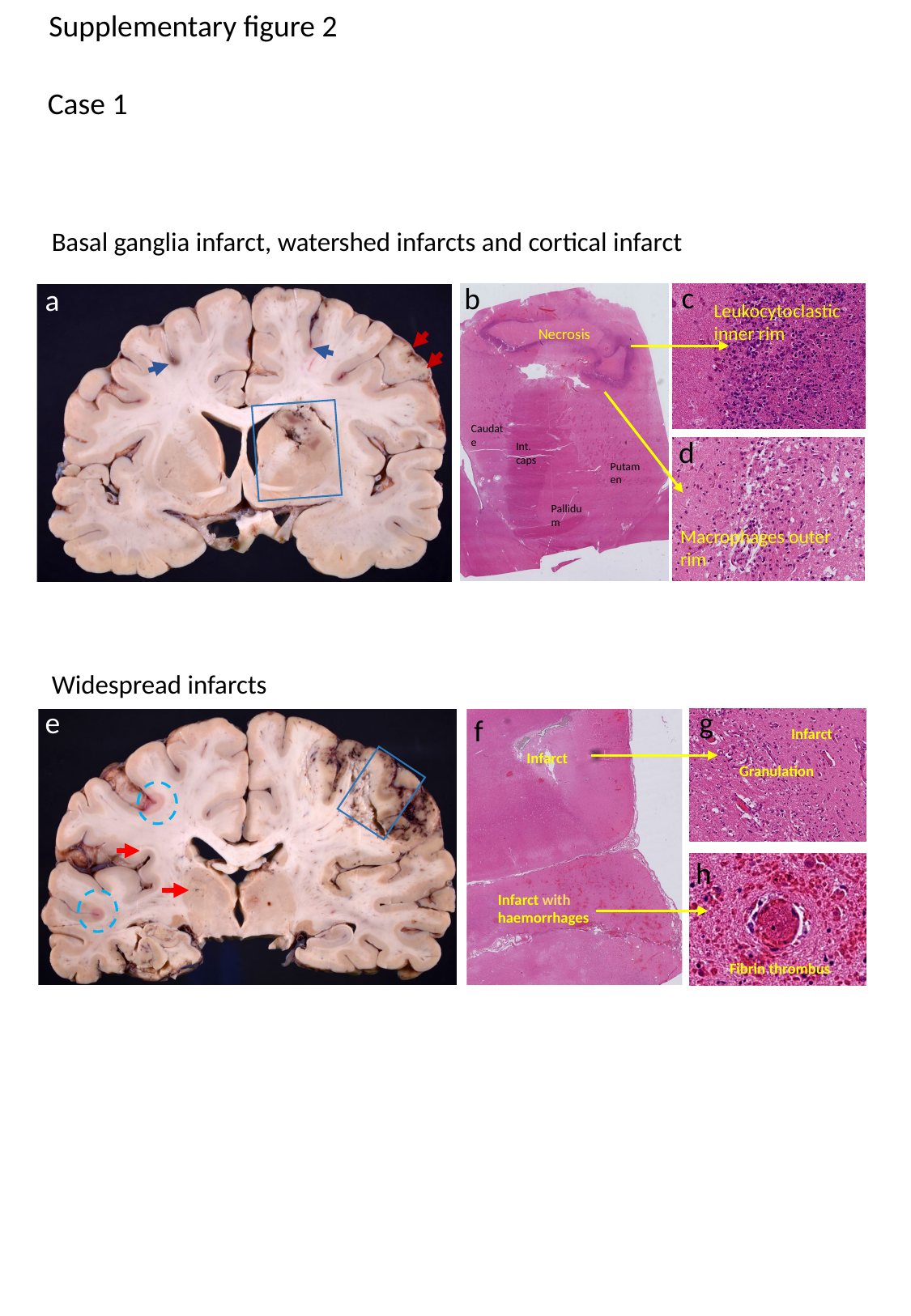

Supplementary figure 2
Case 1
Basal ganglia infarct, watershed infarcts and cortical infarct
c
b
a
Leukocytoclastic inner rim
Necrosis
Caudate
d
Int. caps
Putamen
Pallidum
Macrophages outer rim
Widespread infarcts
e
g
f
Infarct
Infarct
Granulation
Infarct with haemorrhages
Fibrin thrombus
h

## Slide 3
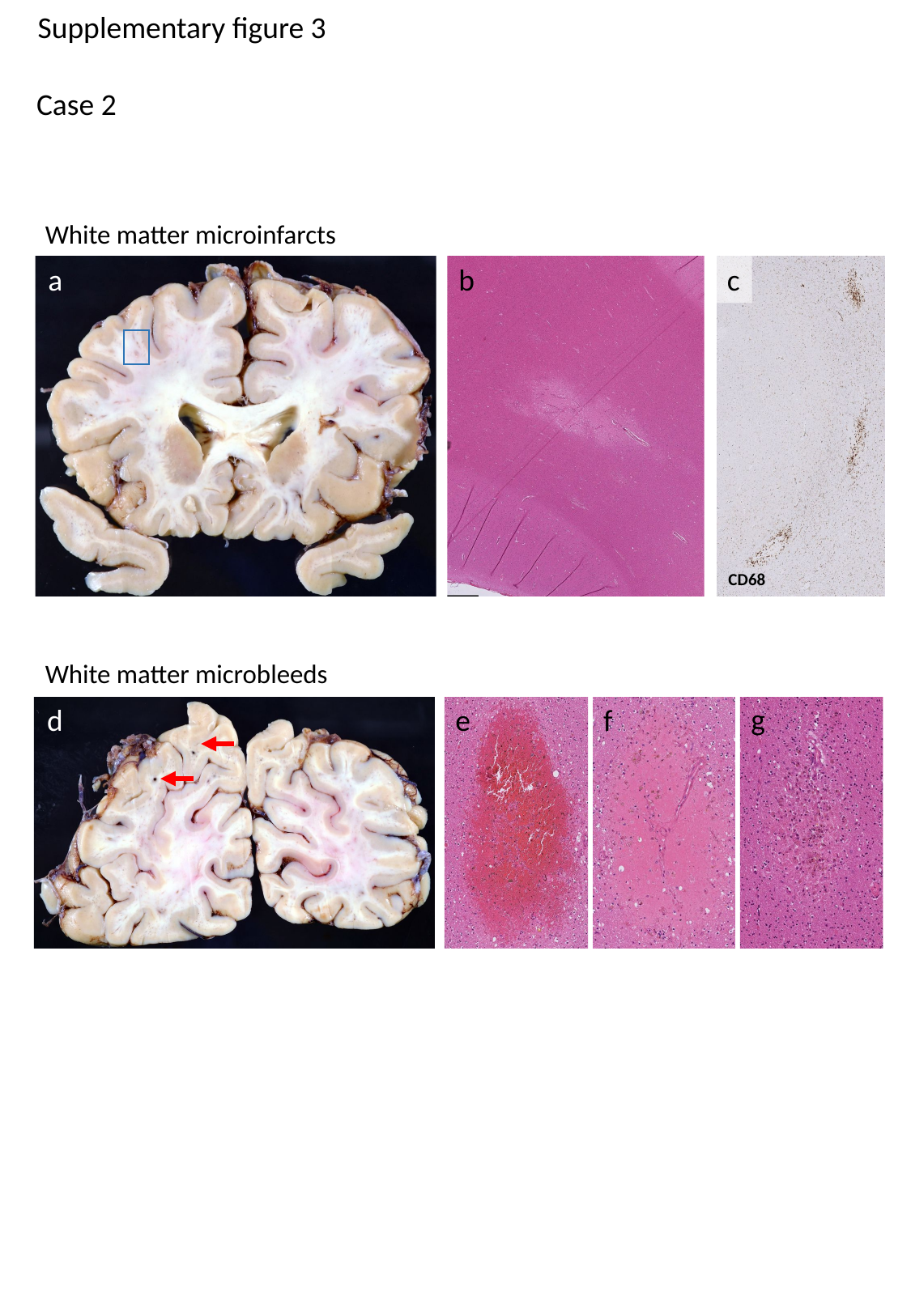

Supplementary figure 3
Case 2
White matter microinfarcts
a
b
c
CD68
White matter microbleeds
g
d
e
f

## Slide 4
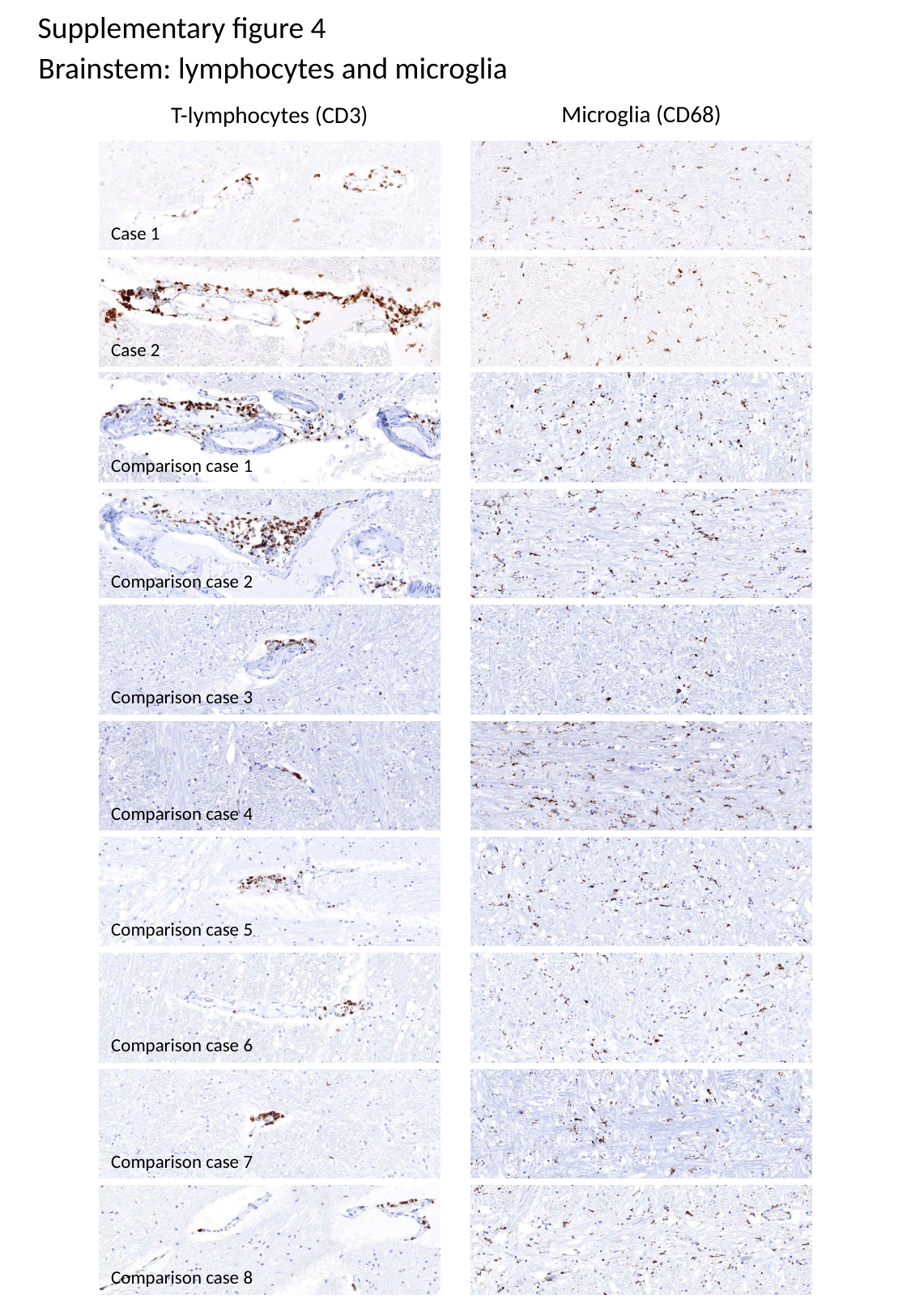

Supplementary figure 4
Brainstem: lymphocytes and microglia
Microglia (CD68)
T-lymphocytes (CD3)
Case 1
Case 2
Comparison case 1
Comparison case 2
Comparison case 3
Comparison case 4
Comparison case 5
Comparison case 6
Comparison case 7
Comparison case 8
