# Supplementary material for: Microvascular injury and hypoxic damage: emerging neuropathological signatures in COVID-19
Source: Acta Neuropathol. 2020 Jul 8;140(3):397–400. doi: 10.1007/s00401-020-02190-2 (PMC7340758; doi:10.1007/s00401-020-02190-2)
Supplement: Supplementary file 2 — Supplementary file2 (DOCX 35 kb) [file 401_2020_2190_MOESM2_ESM.docx]

**Supplementary data:**

**Supplementary figure 1**: Diagram of clinical course of the two patients, with key events before and after admission. The timeline prior to admission indicates weeks, and after admission individual days, with indication of laboratory, imaging and intensive care interventions. 5-day intervals are highlighted in grey.

**a**, Male patient in his early fifties with past medical history of type 2 diabetes. He was admitted to hospital with increasing dyspnoea on the background of cough and fever for two weeks. Due to worsening respiratory symptoms, the patient was intubated and ventilated two days after admission. The patient was considered as a candidate for veno-venous extracorporeal membrane oxygenation (vvECMO) treatment, for persistent hypoxaemia, despite optimal mechanical ventilation. Just prior to vvECMO, the patient went into cardiac arrest and received cardio-pulmonary resuscitation with administration of adrenaline. He had absent-to-low blood flow for 3 minutes. Computed tomography (CT) of the head several hours after vvECMO cannulation showed multifocal cerebral and cerebellar infarcts and CT thorax demonstrated saddle pulmonary embolism. The patient also developed acute kidney injury. vvECMO was discontinued after 4 days, and a repeat CT head 7 days later confirmed further evolution of the multiple infarcts with midline shift and the patient died shortly afterwards.

**b**, Female patient in her mid-sixties with a past medical history of hypertension. She was admitted to hospital in a hypoxic state with reduced consciousness following several days of COVID-19 related symptoms. She was intubated and ventilated on hospitalisation and required renal replacement therapy for acute kidney injury. Importantly, the patient remained unresponsive, despite discontinuation of sedation. Repeat chest X-ray showed signs of viral pneumonia and MRI studies of the brain showed multiple, disseminated small infarcts in the subcortical cerebral white matter as well as microbleeds, an established small infarct in the right thalamus, FLAIR hyperintensity in the right intraparietal sulcus, and bilateral signal changes in cerebellum, suggestive of recent small haemorrhages as well as microbleeds. The patient died 3 weeks after admission due to persistent multiorgan failure.

CT= computed tomography; CAP, chest abdomen pelvis; XR, X-ray; RRT, renal replacement therapy; vvECMO, veno-venous extracorporeal membrane oxygenation; CRP, C reactive protein; CRP, cardio-pulmonary resuscitation; GCS, Glasgow coma scale.

**Supplementary figure 2**: Main macroscopic and microscopic findings of case 1. **a** Infarct in the right caudate, internal capsule and putamen (frame corresponds to **b**), bilateral watershed infarcts in the anterior cerebral artery (ACA) and middle cerebral artery (MCA) territories (blue arrows) and right frontal infarct within the middle cerebral artery territory (red arrows). **b**, Histology of the basal ganglia infarct with striking demarcation of a leukocytoclastic inner rim shown in **c** at a high magnification. **d** The outer rim of granulation tissue and macrophages corresponds to the typical picture of the demarcation zone of a chronic infarct. **e** Multiple infarcts in the right MCA territory (frame corresponds to **f**), watershed infarcts on the left (ACA-MCA and MCA-PCA territories, blue circles), and left PCA infarcts involving thalamus and hippocampus, red arrows). **f** Histology of the framed area shown in **e** with a classical ischaemic infarct (upper part) and an infarct with frequent thrombosed vessels with perivascular haemorrhages (lower part). Both areas are shown at higher magnification in **g** and **h**.

**Supplementary figure 3**: Main macroscopic and microscopic findings of case 2. **a** Some of the subacute white matter microlesions are seen as grey discoloured foci (frame in the left frontal lobe is around one of the microlesions, which is shown on a higher power in **b**). **b** This lesion corresponds to a classic small ischaemic infarct. **c** Immunostaining for CD68 highlights many more microvascular lesions in the subcortical white matter. **d** Particularly in the occipital lobes, there are microbleeds in the subcortical white matter (red arrows). The morphology of the microbleeds varies from **e** fresh haemorrhages, **f** subacute haemorrhages, **g** to haemosiderin-laden macrophage rich microvascular lesions. Also present (not shown) is hyaline arteriolosclerosis in the white matter and atherosclerosis in the blood vessels at the base of the brain.

**Supplementary figure 4**: Perivascular loose T lymphocyte infiltrates and up to moderate microglial activation are not a distinctive feature in the brainstem of COVID-19 patients. Left: In case 1 and 2, there are variably frequent loosely arranged perivascular T lymphocytes, which to a similar extent and density also are present in patients with varied clinical presentations and neuropathological findings. Right: Likewise, there is up to moderately dense diffuse microglial activation without any microglial nodules in cases 1 and 2, and microglial activation to a similar extent and density is a typical post-mortem finding in patients with varied neurological diseases. In both index cases, there was no noticeable B-lymphocyte infiltration. All comparison medulla regions are from patients who died before the COVID-19 pandemic (pre-2020). The age ranges, sex, clinical diagnosis and main neuropathology findings in these patients are listed in supplementary table 1.

**Supplementary table 1:**

| **Comparison case No** | **Age** | **Sex** | **Clinical diagnosis** | **Main neuropathology** |
| --- | --- | --- | --- | --- |
| 1 | 80-85 | M | Several year-long history of dementia with rapid deterioration | Acute microbleed in thalamus and intermediate level Alzheimer’s disease pathological change |
| 2 | 75-80 | M | Rapid cognitive decline | Progressive multifocal leukoencephalopathy |
| 3 | 80-85 | M | Confusion, dysphagia | Multiple microinfarcts in cerebral cortex, intermediate level Alzheimer’s disease pathological change |
| 4 | 55-60 | M | Rapid neurological decline following nausea and dizziness | Meningoencephalitis |
| 5 | 75-80 | M | Multiple chronic systemic diseases, ongoing seizures following myocardial infarction | Hypoxic/ischaemic brain damage, microinfarcts and microbleeds in neocortex, Lewy body pathology |
| 6 | 90-95 | M | Acute neurological decline | Abscess in forebrain |
| 7 | 75-80 | M | Progressive encephalopathy and neurological decline following fever | Intermediate level Alzheimer’s disease pathological change, limbic TDP43 pathology |
| 8 | 85-90 | F | Rapid neurological decline following a fall | Low level Alzheimer’s disease pathological change, limbic TDP43 pathology |
